# Can you tell people’s cognitive ability level from their response patterns in questionnaires?

**DOI:** 10.3758/s13428-024-02388-2

**Published:** 2024-03-25

**Authors:** Stefan Schneider, Raymond Hernandez, Doerte U. Junghaenel, Haomiao Jin, Pey-Jiuan Lee, Hongxin Gao, Danny Maupin, Bart Orriens, Erik Meijer, Arthur A. Stone

**Affiliations:** 1 Dornsife Center for Self-Report Science, and Center for Economic & Social Research, University of Southern California, 635 Downey Way, Los Angeles, CA 90089-3332 USA; 2 Department of Psychology, University of Southern California, Los Angeles, CA USA; 3 Leonard Davis School of Gerontology, University of Southern California, Los Angeles, CA USA; 4 School of Health Sciences, Faculty of Health and Medical Sciences, University of Surrey, Guildford, UK; 5 Center for Economic and Social Research, University of Southern California, Los Angeles, CA USA

**Keywords:** Cognitive ability, Questionnaire responding, Item response theory, Worst performance rule, Task complexity

## Abstract

Questionnaires are ever present in survey research. In this study, we examined whether an indirect indicator of general cognitive ability could be developed based on response patterns in questionnaires. We drew on two established phenomena characterizing connections between cognitive ability and people’s performance on basic cognitive tasks, and examined whether they apply to questionnaires responses. (1) The worst performance rule (WPR) states that people’s worst performance on multiple sequential tasks is more indicative of their cognitive ability than their average or best performance. (2) The task complexity hypothesis (TCH) suggests that relationships between cognitive ability and performance increase with task complexity. We conceptualized items of a questionnaire as a series of cognitively demanding tasks. A graded response model was used to estimate respondents’ performance for each item based on the difference between the observed and model-predicted response (“response error” scores). Analyzing data from 102 items (21 questionnaires) collected from a large-scale nationally representative sample of people aged 50+ years, we found robust associations of cognitive ability with a person’s largest but not with their smallest response error scores (supporting the WPR), and stronger associations of cognitive ability with response errors for more complex than for less complex questions (supporting the TCH). Results replicated across two independent samples and six assessment waves. A latent variable of response errors estimated for the most complex items correlated .50 with a latent cognitive ability factor, suggesting that response patterns can be utilized to extract a rough indicator of general cognitive ability in survey research.

**Supplementary Information:**

The online version contains supplementary material available at 10.3758/s13428-024-02388-2.

## Introduction

Standardized self-report questionnaires are an ever-present research method in the social and medical sciences. Questionnaires are administered in many cross-sectional and longitudinal studies to collect information on a broad range of topics, including people’s behaviors and feelings, attitudes and opinions, health status, personality, environmental conditions, life events, and well-being (Groth-Marnat, 2003). Compared to other forms of data collection, such as behavioral observations or laboratory-based experiments, an advantage of self-report questionnaires is that they allow researchers to gather information from large numbers of respondents via paper and pencil or over the internet quickly, easily, and inexpensively.

Cognitive capacities are relevant to a wide range of behaviors, experiences, and everyday functions assessed in survey research (Jokela, 2022; Llewellyn et al., 2008). However, standardized cognitive assessments are relatively expensive, burdensome to respondents, and sometimes difficult to implement in large survey research. For this reason, many survey studies do not administer formal tests of cognitive abilities alongside questionnaires. In this study, we ask whether there are alternatives to formal cognitive tests that could be used to infer people’s cognitive ability levels from their behaviors in self-report surveys.

It is widely acknowledged that completing self-report questionnaires is a cognitively demanding task that involves various mental processes, including perception, attention, decision-making, and executive control. For each question in a survey, respondents need to read and understand the question, search the relevant information from memory, integrate the information into a summary judgment, and select a response that best reflects their judgment based on the retrieved information (Tourangeau, 1984, 2018), paralleling steps that are assumed to govern many cognitive problem-solving tasks (e.g., encoding, inference, mapping, application, justification, response; Sternberg, 1979). If objective indicators could be developed based on people’s response patterns that reflect *how well* they perform at the task of completing questionnaires, then an indirect measurement of cognitive ability would become available as a by-product of a survey. This could provide an opportunity to measure cognitive abilities from questionnaires that are already administered as part of a survey study without any additional costs or respondent burden.

## Prior research relating questionnaire response quality to cognitive ability

Several previous studies have provided evidence for empirical relationships between people’s cognitive abilities and the quality of their questionnaire responses. For example, respondents with lower cognitive abilities have been found to show more acquiescent responses (i.e., “yeah-saying” or agreeing with statements regardless of item content) in personality assessments (Lechner & Rammstedt, 2015; Schneider et al., 2021), to display more extreme response tendencies in emotional well-being assessments (Schneider, 2018), and to provide more indifferent (“don’t know”) responses in population-based surveys (Colsher & Wallace, 1989; Knäuper et al., 1997). Moreover, lower cognitive abilities have been associated with a greater tendency to provide conflicting answers to similar questions (Colsher & Wallace, 1989), with greater response outliers (Schneider et al., 2022, 2021), and with more random (e.g., internally inconsistent) response patterns (Conijn et al., 2020; Schneider et al., 2022).

## Challenges with using questionnaire response patterns as cognitive ability indicators

Even though these studies support the idea that the quality of people’s responses in questionnaires may at least partially reflect their cognitive capacity, to our knowledge, there have not been any formal attempts to use response patterns in questionnaires as an indirect measure of cognitive ability. Indeed, there are several significant challenges that make it difficult to unambiguously link questionnaire response patterns to cognitive ability levels. First, whereas standardized cognitive tests of intelligence or ability level are often constructed to assess the number of test items a person solves correctly (Kyllonen & Zu, 2016), many self-report questionnaires are intended to measure subjective attitudes and experiences, and there generally are no objectively correct and incorrect answers to these questions. In the absence of a “ground truth” for response accuracy, attempts to measure cognitive abilities from the quality of responses in questionnaires, therefore, must typically rely on psychometric indicators that quantify presumable “abnormalities” in response patterns, such as the extent to which a response is improbable given other information, inconsistent with other responses, or deviating from statistically expected responses (Schneider et al., 2022).

Second, many factors can influence observed response patterns, and it is usually impossible to attribute a pattern uniquely to a particular causal factor. For example, a low-quality answer pattern might reflect low cognitive ability or it might reflect carelessness, such that a respondent is not motivated enough to read the questions or does not attempt to find answers that reflect his or her true attitudes or opinions (Ward & Meade, 2023). Responses of lower quality could also result when a participant is distracted for a moment, gets fatigued when answering long questionnaire batteries, or is unfamiliar with the topic asked in the questions (Krosnick, 1991). These ambiguities are not unique to the measurement of cognitive abilities from questionnaire responses, and they similarly apply to other indirect indicators of cognitive ability such as response times on reaction time tasks (Kyllonen & Zu, 2016).

Third, even though prior research has documented consistent relationships between questionnaire response patterns and respondents’ cognitive ability, the strength of these relationships has generally been modest. The magnitude of the observed associations resembles the reliable, but not impressively high, correlations that have been observed between reaction times and measures of general intelligence (Schmiedek et al., 2007). To date, there have been no concentrated efforts to examine whether the signal that links questionnaire response patterns with cognitive ability can be meaningfully enhanced.

## The present study

The goal of this study is to more firmly establish evidence supporting the idea that response patterns in questionnaires can be potentially useful as indirect indicators of cognitive ability, and to investigate which information from questionnaire response patterns is most closely associated with people’s cognitive ability levels. To do this, we draw on two key findings in intelligence research that have been reliably documented to characterize connections between general cognitive ability and people’s performance on elementary cognitive tasks (e.g., simple choice tasks): the *worst performance rule* (WPR) and the *task complexity hypothesis* (TCH).

The WPR states that people’s worst performance on multiple sequential tasks is more indicative of their cognitive ability than their average or best performance (Larson & Alderton, 1990). The most prominent theoretical explanation for this phenomenon is based on the idea that temporary lapses of attention or working memory lead to momentarily poor performance and that individuals of higher general cognitive ability have a better capacity for attentional control, thus preventing such lapses (Jensen, 1992; Larson & Alderton, 1990).

The TCH suggests that people’s performance on more complex versions of an elementary cognitive task correlates more highly with general cognitive abilities than less complex versions of the same task, presumably due to greater “g saturation” of more complex tasks (Kranzler et al., 1994; Larson et al., 1988; Stankov, 2000). The importance of the TCH for information processing is emphasized by Jensen (2006), who considered the observation that “individual differences in learning and performance increase monotonically as a function of increasing task complexity” (p. 205) as so central to label it the first law of individual differences.

Even though the predictions by the WPR and TCH have been most prominently studied in the measurement of cognitive processing speed by means of reaction times (i.e., “mental chronometry”), they have been viewed as universal and key phenomena that any process theory of cognitive ability has to account for (Jensen, 2006; Schubert, 2019). Thus, we argue that if response patterns on questionnaires reveal information about individual differences in general cognitive ability, they should also follow the WPR and TCH.

To test the predictions of the WPR and TCH, we conceptualize each item of a self-report questionnaire as a separate task and utilize a psychometric procedure based on item response theory (IRT) to capture the quality of responses on an item-by-item basis for each person. Obtaining an indicator of response quality separately for each questionnaire item is necessary in order to distinguish for which items an individual performed better versus worse (to test the WPR) and to evaluate people’s performance on less versus more complex items (to test the TCH). Specifically, we propose to measure item response quality as the extent to which the selected response differs from the statistically expected response given an IRT model, that is, the estimated response “*error*” score for each item. Following the prediction of the WPR, we hypothesize that the largest response error scores for each person reflect their worst performance in a self-report survey and are, therefore, most highly correlated with their general cognitive ability. Following the prediction of the TCH, we hypothesize that response error scores for more complex questionnaire items (i.e., items with a greater information load; Jensen, 2006) are more strongly associated with cognitive ability than response error scores for less complex questions. Finally, we investigate to what extent an indirect measurement of cognitive ability derived from response patterns in questionnaires can be meaningfully enhanced by taking the complexity of survey items into account. To evaluate the robustness of the results, we examine their replicability across two independent samples and across multiple measurement waves using survey data from a large US population-based panel study.

## Methods

### Study sample

The sample for the present analyses was drawn from the Health and Retirement Study (HRS), a longitudinal study that has been collecting data on the health, social, and economic well-being of older Americans since 1992 (Juster & Suzman, 1995). HRS participants are interviewed in two-year intervals. Initial HRS respondents were individuals between 51 and 61 years of age and their spouses. New cohorts were added over time to render the participant sample representative of the US population 50 years and older (Sonnega et al., 2014).

Since 2006, the HRS has administered the Participant Lifestyle Questionnaire (PLQ), also referred to as “leave-behind survey” (Smith et al., 2013). This self-administered questionnaire package was handed to respondents following the core interview, to be returned by mail. The PLQ has been administered every two years to a rotating 50% randomly selected subsample of participants who completed the core interviews. Half of the participants were assigned to complete the PLQ in Waves 8, 10, and 12, and the remaining (nonoverlapping) 50% were assigned to complete it in Waves 9, 11, 13, and so on. For the present study, we included the Wave 8 subsample (PLQ administered in 2006) as the primary analysis sample. We repeated the analyses in the Wave 9 subsample (PLQ administered in 2008) to examine the replicability of findings in an independent sample. In secondary analyses shown in the online appendix, we also repeated the analyses for subsequent Waves 10 to 13 to examine the robustness and replicability of results over time and with repeated assessments. We included only respondents who completed the PLQ by themselves; individuals whose questionnaires were completed by proxy respondents (between 1% and 2% per wave) were not included. All participants provided informed consent as part of the HRS, and the research was approved by the relevant institutional review boards.

Demographic characteristics of the analyzed Wave 8 and Wave 9 HRS samples are shown in Table 1. A total of *n* = 7296 (Wave 8) and *n* = 6646 (Wave 9) participants were analyzed. Participants’ mean ages were 68.7 years (*SD* = 10.0, Wave 8) and 69.9 years (*SD* = 9.8, Wave 9). About three fifths of participants in each sample were female, four fifths were White, and about three fifths were married. Participants had on average completed 12.6 years of education, and the median income was about $37,000.

**Table 1:** Sample characteristics

	Mean (*SD*) or frequency (%)	
Characteristic	Wave 8 sample	Wave 9 sample
	(*N* = 7296)	(*N* = 6646) ^a^
Age	68.65 (10.04)	69.92 (9.76)
Female	4284 (58.72%)	4007 (60.29%)
Race		
White	6054 (82.98%)	5514 (82.98%)
African American	950 (13.02%)	844 (12.70%)
Other	292 (4.00%)	287 (4.32%)
Hispanic ethnicity	534 (7.32%)	570 (8.58%)
Married	4706 (64.50%)	4029 (60.63%)
Years of education	12.64 (3.06)	12.60 (3.10)
Highest educational degree		
Less than high school	1355 (18.57%)	1324 (19.92%)
High school	2702 (37.05%)	2403 (36.16%)
Some college	1641 (22.49%)	1507 (22.68%)
College graduate or higher	1597 (21.89%)	1411 (21.23%)
Income		
Less than $25,000	2319 (31.78%)	2139 (32.18%)
$25,000 to $49,999	2061 (28.25%)	1865 (28.06%)
$50,000 to $74,999	1116 (15.30%)	1024 (15.41%)
$75,000 to $99,999	650 (8.91%)	555 (8.35%)
$100,000 and above	1150 (15.76%)	1063 (15.99%)
Cognitive ability sum score (0–27)	15.26 (4.35)	15.13 (4.33)

^a ^*N* = 6645 for race, ethnicity, marital status, and educational degree

The analyses excluded respondents from the larger HRS cohorts who had participated in the core interview but did not return the paper-and-pencil PLQ by mail or did not complete the PLQ by themselves. This excluded *n* = 1129 (13.40%, Wave 8) and *n* = 1487 (18.28%, Wave 9) HRS respondents. Compared with the analysis samples, excluded HRS respondents were on average (across both waves) 1.92 years older, were 5.65% less likely women, 12.03% less likely White, and 8.07% less likely married, had 1.12 fewer years of education, and had $10,000 lower median income.

### Cognitive ability measurement

Participants’ cognitive ability was measured with a composite of five standardized cognitive tests administered in the HRS to each participant at each wave. The test battery is based on the widely used Telephone Interview for Cognitive Status (Ofstedal et al., 2005) and it includes immediate free recall (0–10 points) and delayed free recall (0–10 points) to measure memory, a serial sevens subtraction test to measure attention and working memory (0–5 points), and backward counting from 20 to measure general mental processing (0–2 points). We followed previous studies (Crimmins et al., 2016) for calculating an overall cognitive ability score by summing the scores for all five subtests (possible score range: 0–27). A small percentage of participants (0.8–3.1%) did not provide scores for immediate and delayed free recall and serial sevens subtraction tests. HRS has developed an imputation algorithm for cognitive variables for all waves (McCammon et al., 2019), and we used the imputed data to accommodate missing scores on subtests. The overall cognitive ability score had mean = 15.26, *SD* = 4.35, composite reliability omega = .73 at Wave 8, and mean = 15.13, *SD* = 4.33, omega = .74 at Wave 9.

### Self-report questionnaires

We analyzed responses for 102 self-report questions included in 21 multi-item psychometric rating scales assessed in the HRS PLQ. We only analyzed responses to questionnaires that were applicable to all respondents (i.e., questionnaires on participants’ experiences with their spouse, children, or work environment that were relevant only to respondent subgroups were excluded). Specifically, the constructs addressed by the questionnaires were life satisfaction (5 items), cynical hostility (5 items), optimism (6 items), hopelessness (4 items), loneliness (3 items), neighborhood physical disorder (4 items), neighborhood social cohesion (4 items), constraints on personal control (5 items), perceived mastery (5 items), religiosity/spirituality (4 items), everyday discrimination (5 items), social effort/reward balance (3 items), neuroticism (4 items), extraversion (5 items), conscientiousness (5 items), agreeableness (5 items), openness to experience (7 items), purpose in life (7 items), anxiety (5 items), anger-in (4 items), and anger-out (7 items). All items were rated on ordinal rating scales (e.g., strongly agree – strongly disagree) with between three and seven response options. For details on the questionnaires and their psychometric properties, see Smith et al. (2013).

### Indicators of question complexity

Following Jensen (2006), we refer to the term complexity as the information load involved in answering a self-report question. Information load cannot be assessed with a single attribute, and we coded 10 different characteristics of each question that are likely related to information load based on prior literature (Bais et al., 2019; Knäuper et al., 1997; Schneider, Jin et al., 2023a; Yan & Tourangeau, 2008) using four approaches, as described below.

#### Indicator 1

We counted the number of words in each item as a simple indicator of the number of task elements a respondent needed to attend to when answering the question. A binary word count (WC) variable was then created based on a median split of the observed item word counts: Items that consisted of 10 or more words were categorized as “longer” items, and items with fewer than 10 words were categorized as “shorter” items.

#### Indicator 2

Questions requiring greater verbal ability were coded using the Dale–Chall (DC) word list. The list contains approximately 3000 words that fourth grade students can generally reliably understand (Chall & Dale, 1995). Based on a median split across all items, we coded items containing two or more words that were not on the Dale–Chall word list as requiring greater verbal ability, and items with all words on the list or only one word not on the list as requiring less verbal ability.

#### Indicators 3 to 5

The Question Understanding Aid (QUAID; http://quaid.cohmetrix.com/), an online software tool for survey developers, was used to identify item wordings that may reduce the clarity of survey questions and increase the degree of uncertainty about the required response. The validity and utility of the QUAID has been previously established (Graesser et al., 2006; Graesser et al., 2000). Each item was categorized based on three types of potentially problematic wordings: presence or absence of (3) unfamiliar technical terms (UTT), (4) vague or imprecise relative terms (VRT), and (5) vague or ambiguous noun phrases (VNP).

#### Indicators 6 to 10

The Linguistic Inquiry and Word Count program (LIWC; Pennebaker et al., 2015) was used to identify item wordings that increase the degree of stimulus discrimination involved in reading and understanding a survey question. For each item, we coded whether or not it contained any (6) conjunctions (CON; e.g., if, whereas, because), (7) negations (NEG; e.g., no, not, never), (8) words indicating discrepancies (DIS; e.g., should, would, could), (9) words indicating tentative statements (TEN; e.g., maybe, perhaps), and (10) words indicating differentiation or exclusion (EXC; e.g., has not, but, else).

A composite measure of item complexity was computed by taking the sum of all 10 individual complexity indicators (possible range of the composite measure = 0 to 10).

### Data analysis

We first describe the statistical methods to derive the proposed indicator of response quality (i.e., estimated response “error” scores) for the questionnaire item responses. Subsequently, we describe the analysis strategy to test the predictions of the WPR and TCH.

#### Response error scores derived from item response theory model

Whereas multi-item rating scales are typically used in research to estimate people’s actual scores on the underlying construct targeted by each scale (e.g., optimism, personality traits), our goal was to develop indicators that captured *how closely* people’s responses *on each item* reflected their presumable true scores on the underlying constructs. We used an IRT framework to estimate these true scores, which then served as a reference point for estimating response error scores for each item.

In a first modeling step, we fitted a unidimensional graded response model (GRM; Samejima, 1969) to each of the 21 PLQ scales at each assessment wave to estimate people’s presumable true scores for each of the scales. The GRM is a popular and flexible IRT model for ordered categorical responses. Let θ be the latent construct underlying the responses to the items in a scale, and suppose the items have *m* ordered response options. Let P*_ijk_(*θ*) be the probability that the *i*th respondent with a latent score *θ*_*i*_ on the construct chooses response category *k* or higher on the *j*th item of the scale. The GRM then specifies P*_ijk_(*θ*) as a monotonically increasing function of the latent score *θ*:

1 $${P}_{ijk}^{*}=P\left({Y}_{ij}\ge k\left|{\theta }_{i},{a}_{j},{b}_{jk}\right.\right)$$

where *Y*_*ij*_ is the item response of person *i* to item *j* (responses can take values of 1, 2, . . . , *m*_*j*_), *a*_*j*_ is the item discrimination parameter, and *b*_*jk*_(*k =* 1, 2, . . . , *m*_*j*_ - 1) are threshold parameters for item *j* that separate two adjacent response categories *k* and *k +* 1. The probability of choosing a particular response category *k* at a given level of *θ* is given by the item category response function:

2 $${P}_{ijk}\left(\theta \right)={P}_{ijk}^{*}\left(\theta \right)-{P}_{ij\left(k+1\right)}^{*}\left(\theta \right)$$

The GRM allows for item discrimination and threshold parameters to differ across items. The discrimination parameter indicates the strength of the relationship between an item and the measured construct. The threshold parameters provide information on the extent to which an item targets higher or lower levels of the construct (e.g., the “severity” level of a problem addressed by the question). For each of the 21 PLQ scales, we evaluated the fit of a unidimensional GRM by means of the comparative fit index (CFI >.95 for good model fit), Tucker–Lewis index (TLI >.95 for good fit), root mean square error of approximation (RMSEA < .06 for good fit), and standardized root mean square residual (SRMR < .08 for good fit). Across scales, the average model fit values were CFI = .970 (range = .868–1.00), TLI = .942 (range = .790–1.00), RMSEA = .104 (range = .000–.237), and SRMR = .038 (range = .000–.152) in the Wave 8 sample, and CFI = .969 (range = .879–1.00), TLI = .941 (range = .799–1.00), RMSEA = .116 (range = .000–.226), and SRMR = .041 (range = .000–.139) in the Wave 9 sample. For details, see Table S1 in the online Supplemental Appendix.

In a second step, we obtained latent variable estimates ($\widehat{\theta }$) of each person’s “true” scores on the underlying constructs using expected a posteriori parameter estimation, and calculated the response error scores by comparing the observed response for each item with the statistically expected response. The expected response was calculated as the weighted sum of the probabilities of all response categories given the person’s level of $\widehat{\theta }$, where the weights represent the response category value (e.g., values 1 to 5 on a five-point rating scale):

3 $$E\left(Y\right|\widehat{\theta })= \sum_{k=1}^{m}kP(Y=k\left|\widehat{\theta }\right).$$

we then calculated response error scores in the following form:

4 $$response\;error\left(\widehat{\theta }\right)=\frac{\left| y- E\left(Y\right|\widehat{\theta }\right) |}{m-1}.$$

The numerator is the absolute value of the usual residual term, that is, the absolute difference between observed score *y* and the expected score. The absolute value of the difference was used because we were interested only in the magnitude of the residual, and not its direction, as an indicator of response quality. For an individual giving optimal responses, the observed score should be close to the expected score predicted by the model. Larger absolute deviations from the expected score are indicative of low response quality. The denominator in Eq. 4 rescales the values of the absolute residuals by the number of threshold parameters (i.e., number of response categories *m* minus 1*)* for a given item. For each response, the resulting values of the response error scores can range from 0 (i.e., the response perfectly matches the statistically expected response) to 1 (the observed response is maximally different from the expected response). Response error scores were computed from all scales with no missing responses per person; the overall rate of missing item responses was 2.98% in the Wave 8 sample and 2.80% in Wave 9 sample.

#### Analysis of worst performance rule

To test the prediction of the WPR, we first divided each participant’s response error scores for all questionnaire items into deciles such that the first category comprised the 10% smallest error scores and the 10th category contained the 10% largest error scores for each person. We then averaged the error scores in each decile so that each participant had 10 mean error scores (one per decile) and estimated the correlation between people’s cognitive test scores and their mean error scores in each decile. According to the WPR, the correlations should differ in magnitude from the lowest to the highest decile. To test this, we needed to consider that the correlations were nonindependent (i.e., correlated). Accordingly, we estimated a correlation matrix consisting of the correlations among the mean error scores for each decile and their corresponding correlations with the cognitive test scores. We then Fisher *z*-transformed the correlations and conducted an omnibus Wald test examining whether the (dependent) correlations between the mean error scores and cognitive ability scores differed across the 10 deciles. Significant omnibus tests were followed by post hoc comparisons between correlation pairs conducted using the delta method.

#### Analysis of task complexity hypothesis

Multilevel regression models were estimated to test the TCH. The respondents’ response error scores for all 102 items (nested in individuals) served as outcome variable in the multilevel models. The error scores were regressed on item complexity scores at Level 1, allowing for random intercepts and random regression slopes across individuals, and the intercepts and slopes were regressed on cognitive test scores at Level 2. For respondent *i* and item *j*, the model equation was as follows:

- Level 1:
  $$response\;{error}_{ij}={\beta }_{0i}+{\beta }_{1i}complexity+{r}_{ij}$$
- Level 2:
  5 $$\begin{array}{l}{\beta }_{0i}={\gamma }_{00}+{\gamma }_{01}ability+{u}_{0i}\\ {\beta }_{1i}={\gamma }_{10}+{\gamma }_{11}ability+{u}_{1i}\end{array}$$

where $${r}_{ij}\sim N\left(0, {\sigma }^{2}\right)$$ and $$\left(\begin{array}{c}{u}_{0i} \\ {u}_{1i} \end{array}\right)\sim MVN\left(\left(\begin{array}{c}0\\ 0\end{array}\right),\left(\begin{array}{cc}{\tau }_{00}& \\ {\tau }_{10}& {\tau }_{11}\end{array}\right)\right)$$

The reduced-form equation of the same model is as follows:

6 $$response\;{error}_{ij}={\gamma }_{00}+{\gamma }_{01}{ability}_{i}+{\gamma }_{10}{complexity}_{ij}+{\gamma }_{11}{complexity}_{ij}\times {ability}_{i}+{u}_{0j}+{u}_{1j}+{r}_{ij}$$

The item complexity by cognitive ability cross-level interaction term tests the prediction by the THC. A significant interaction indicates that the relationship between cognitive ability and the response error scores depends on item complexity. The primary multilevel models were tested using the composite measure of item complexity (i.e., the sum of all complexity indicators). Secondary analyses used each of the individual indicators of item complexity in separate models to examine the robustness of the results across different complexity indicators.

A final set of analyses addressed the question to what extent the correlation between cognitive ability and people’s response error scores would be meaningfully different for surveys with less versus more complex items. To examine this, we reparameterized the model in Eq. 5 such that it used neither a traditional intercept nor regression slope, but rather parameters representing individual differences in response errors at lower versus higher item complexity levels (see Singer & Willett, 2003, p. 187):

- Level 1:
  7a $${response\;error}_{ij}={\beta }_{0i}\frac{9-complexity}{9}+{\beta }_{1i}\frac{complexity}{9}+{r}_{ij}$$

At Level 2, the parameter *β*_*0ij*_ represents latent individual differences in response errors for items with low complexity (complexity = 0) and *β*_*1ij*_ represents latent individual differences in response errors for items with higher complexity (complexity = 9; the highest observed score, see Results). The two parameters were allowed to correlate with each other at Level 2 and were simultaneously correlated with people’s cognitive ability scores in the multilevel model:

- Level 2:
  7b $$\begin{array}{l}{\beta }_{0i}={\gamma }_{00}+{u}_{0i}\\ {\beta }_{1i}={\gamma }_{10}+{u}_{1i}\\ {ability}_{i}={\gamma }_{20}+{u}_{2i},\end{array}$$

where $${r}_{ij}\sim N\left(0, {\sigma }^{2}\right)$$ and $$\left(\begin{array}{c}{u}_{0i}\\ {u}_{1i}\\ {u}_{2i}\end{array}\right)\sim MVN\left(\left(\begin{array}{c}0\\ 0\\ 0\end{array}\right),\left(\begin{array}{ccc}{\tau }_{00}& & \\ {\tau }_{10}& {\tau }_{11}& \\ {\tau }_{20}& {\tau }_{21}& {\tau }_{22}\end{array}\right)\right)$$

We also tested the same model but instead of using a cognitive ability sum score, we fitted a latent cognitive ability factor underlying the four cognitive subtests (immediate recall, delayed recall, serial 7s, backward counting). The correlation between this latent cognitive ability factor and people’s latent response errors for more complex items represents our best estimate of the maximal correspondence between people’s response errors in questionnaires and participants’ true cognitive ability level. The model fit of the cognitive ability factor was first evaluated in a separate model; because scores on immediate- and delayed-recall tests are highly correlated, we allowed for a residual correlation between these subtests in the factor model. The cognitive ability factor was then incorporated at Level 2 of a multilevel structural equation model where it was correlated with the random effects of response errors for less complex (complexity = 0) and more complex (complexity = 9) items, respectively.

We used the R package mirt (Chalmers, 2012) for the IRT models used to derive the estimated response error scores. Analyses testing the WPR and THC were conducted in M*plus* version 8.10 (Muthén & Muthén, 2017) via the R package MplusAutomation (Hallquist & Wiley, 2018) and using maximum likelihood parameter estimation with standard errors robust to non-normality.

## Results

### Distribution of estimated response error scores

**Fig. 1:**
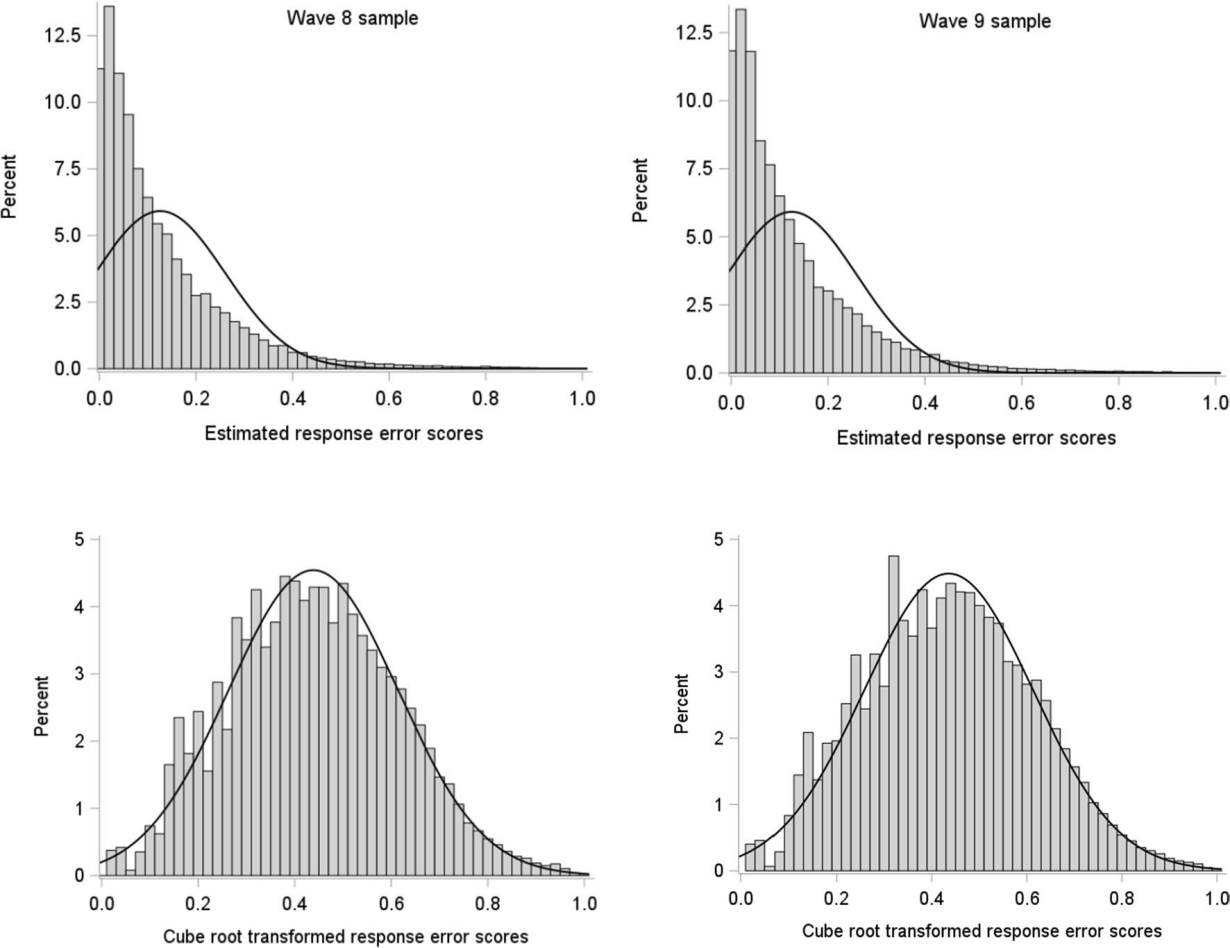
Histograms and normal density curves of estimated response error scores (top) and cube-root-transformed response error scores (bottom) in Wave 8 (left) and Wave 9 (right) samples

The top panel in Fig. 1 shows histograms of the (absolute) response error scores estimated from the GRMs in each sample. Even though the distributions of the estimated response errors covered almost the full possible range, with observed values ranging from <.001 (observed responses nearly exactly matching the expected response) to >.990 (observed responses nearly maximally deviating from the expected response), the distributions were notably positively skewed. The median estimated response errors were .082 (interquartile range [IQR] = .030 to .175; Wave 8 sample) and .081 (IQR = .029 to .175; Wave 9 sample), indicating that the large majority of responses closely matched the statistically expected responses. Because skewed performance distributions have been suggested to distort results from analyses involving the WPR (see Coyle, 2003), we applied a cube root transformation to the response error scores, after which the scores approximated a normal distribution (see Fig. 1, lower panel).

### Worst performance rule

To test the WPR, the transformed response error scores were used to compute mean error scores per respondent decile. As shown in Table 2, the (grand) mean response error scores increased from the lowest (i.e., first) decile (means ranging from .15 to .16 across samples) to the highest (i.e., 10th) decile (mean = .74 in each sample) as expected (i.e., as a logical consequence of the grouping of scores into deciles). The standard deviations of response error scores were comparable across deciles (*SD*s ranging from .04 to .06). As shown in the upper part of Table 2, the correlations between response error scores in different deciles showed a systematic pattern whereby error scores in adjacent deciles were substantially correlated with each other (*r*s ranging between .84 and .97). Error scores at opposite ends of the distribution were only modestly associated with each other (correlations between error scores in first and 10th decile of *r = *.14 in Wave 8 and *r = *.16 in Wave 9 samples, respectively), suggesting that these error scores had the potential to differ in their relationships with cognitive ability.

**Table 2:** Means (SDs) and correlations among mean response error scores across deciles for Wave 8 sample (above main diagonal) and Wave 9 sample (below main diagonal)

	Decile 1	Decile 2	Decile 3	Decile 4	Decile 5	Decile 6	Decile 7	Decile 8	Decile 9	Decile 10
Decile 1	--	.84	.77	.71	.64	.58	.52	.45	.36	.16
Decile 2	.85	--	.94	.88	.82	.75	.69	.62	.51	.28
Decile 3	.77	.95	--	.96	.90	.84	.78	.71	.60	.36
Decile 4	.71	.89	.96	--	.96	.91	.85	.78	.67	.44
Decile 5	.65	.84	.91	.97	--	.97	.91	.84	.75	.52
Decile 6	.59	.78	.85	.91	.97	--	.96	.90	.81	.59
Decile 7	.52	.70	.78	.85	.91	.96	--	.96	.87	.66
Decile 8	.44	.62	.70	.77	.84	.90	.96	--	.94	.73
Decile 9	.34	.51	.59	.67	.74	.81	.87	.94	--	.84
Decile 10	.14	.29	.37	.44	.51	.59	.66	.74	.85	--
Wave 8										
Mean	.16	.25	.31	.37	.41	.46	.51	.56	.63	.74
*SD*	.04	.04	.05	.05	.05	.05	.05	.05	.06	.06
Wave 9										
Mean	.15	.24	.31	.36	.41	.46	.50	.56	.62	.74
*SD*	.04	.05	.05	.05	.05	.05	.05	.06	.06	.06

All correlations are significant at *p* < .001

The correlations between people’s cognitive ability scores and their mean response error scores in each decile are shown in Table 3. Omnibus Wald tests indicated that correlations with cognitive ability differed significantly across the 10 deciles (*p* < .001 in each sample). Response error scores in the first decile (i.e., the smallest response errors) were not significantly correlated with cognitive ability scores (*r* = −.01, *p* = .50 for Wave 8 sample; *r* = −.02, *p* = .16 for Wave 9 sample). Response errors for all other deciles were significantly associated with cognitive ability (*p*s < .001), but the magnitude of the correlations increased monotonically with increasing deciles. Supporting the prediction by the WPR, response error scores in the 10th decile showed the highest correlations with people’s cognitive ability scores (*r* = −.33, *p* < .001 for Wave 8 sample; *r = *−.35, *p* < .001 for Wave 9 sample), significantly exceeding the correlations for all other deciles (*p*s < .01).

**Table 3:** Correlations (95% confidence intervals) between cognitive test scores and mean response error scores across deciles

	Correlation with cognitive test scores	
	Wave 8 sample	Wave 9 sample
Response errors decile 1	−.01 (−.03; .01) ^a^	−.02 (−.04; .01) ^b^
Response errors decile 2	−.05 (−.08; −.03)	−.07 (−.10; −.05)
Response errors decile 3	−.08 (−.11; −.06)	−.10 (−.12; −.08)
Response errors decile 4	−.12 (−.15; −.10)	−.14 (−.17; −.12)
Response errors decile 5	−.16 (−.19; −.14)	−.18 (−.20; −.16)
Response errors decile 6	−.20 (−.23; −.18)	−.22 (−.24; −.20)
Response errors decile 7	−.23 (−.26; −.21)	−.25 (−.27; −.22)
Response errors decile 8	−.27 (−.29; −.25)	−.29 (−.31; −.27)
Response errors decile 9	−.31 (−.33; −.28)	−.32 (−.34; −.30) ^c^
Response errors decile 10	−.33 (−.35; −.31)	−.35 (−.37; −.32) ^c^
Wald test for differences in correlations across all deciles	χ^2^(*df* = 9) = 526.06 *p* < .001	χ^2^(*df* = 9) = 482.75 *p* < .001

All correlation coefficients are significant (*p* < .001) except ^a ^*p* = .50 and ^b ^*p* = .16. All correlations significantly differ from each other between all deciles in each sample (*p* < .001, except ^c ^*p* = .009)

### Task complexity hypothesis

Descriptive statistics for the various indicators of item complexity are shown in Table 4. The occurrence of different aspects of item complexity across the 102 analyzed PLQ questions ranged from 15.7% for items containing negations to 50.0% for items with a word count of 10 or more words. With the exception of the indicator of unfamiliar technical terms, which showed small negative correlations with most other indicators, all item complexity indicators were positively intercorrelated (*r*s ranging from .02 to .66; median *r* = .28), which means that different aspects of complexity tended to co-occur for a given item. A one-factor model for binary indicators showed the following fit: χ^2^ [*df* = 35] = 53.36, *p* = .02; CFI = .971, TLI = .963, RMSEA = .072, SRMR = .123. A composite measure of item complexity created as the sum of all binary indicators had a reliability of categorical omega = .82 (Green & Yang, 2009). The mean score of the composite complexity measure was 3.25 (SD = 2.65; median = 4.00), with a range of 0 to 9 (no item received the maximum possible complexity score of 10).

**Table 4:** Correlations between indicators of item complexity

	Correlation coefficient									
Item complexity indicator	(1)	(2)	(3)	(4)	(5)	(6)	(7)	(8)	(9)	Percent items
(1) Word count ≥10 words	--									50.0%
(2) >1 word not in Dale–Chall word list	.54	--								40.2%
(3) QUAID unfamiliar technical terms	−.02	.14	--							24.5%
(4) QUAID vague or imprecise relative terms	.42	.47	−.03	--						38.2%
(5) QUAID vague or ambiguous noun phrases	.30	.10	.12	.27	--					19.6%
(6) LIWC conjunctions	.47	.34	.00	.20	.24	--				36.3%
(7) LIWC negations	.32	.20	.00	.16	.06	.24	--			15.7%
(8) LIWC discrepancies	.23	.02	−.20	.28	.19	.21	.27	--		23.5%
(9) LIWC tentative statements	.51	.34	−.08	.46	.27	.54	.17	.31	--	43.1%
(10) LIWC differentiation/exclusion	.52	.29	−.03	.20	.22	.61	.43	.23	.66	34.3%

Number of items = 102 for all correlations. Correlation coefficients of ±.20 or higher in magnitude are significant at *p* < .05

**Fig. 2:**
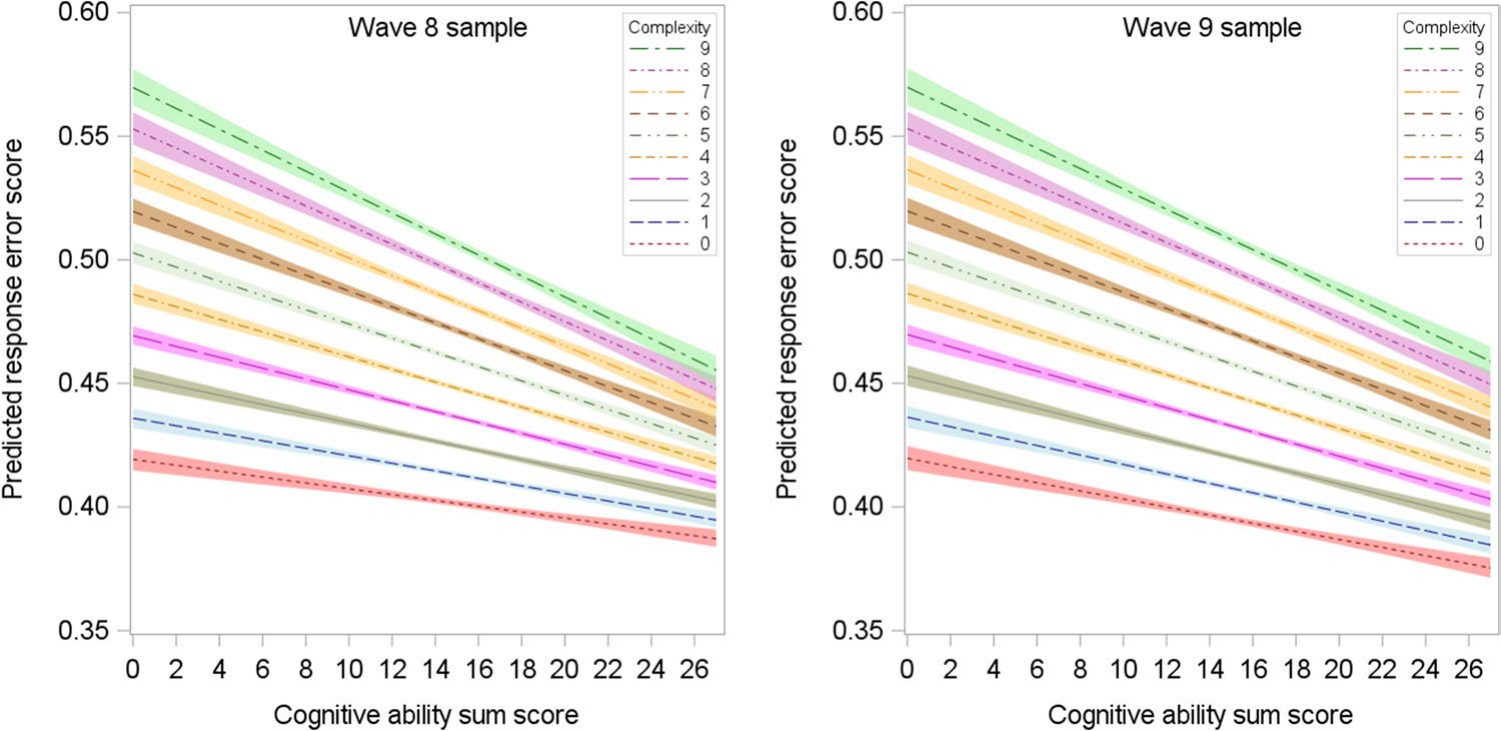
Relationship between cognitive ability sum scores and predicted response error scores by item complexity composite scores in the Wave 8 (left) and Wave 9 (right) samples. Colored bands represent 95% confidence intervals

Results for the moderated multilevel regression models predicting people’s (cube-root-transformed) response error scores from the interaction between their cognitive test scores and item complexity are shown in Table 5 (Wave 8 sample) and Table 6 (Wave 9 sample). The first column in each table presents results for the composite measure of item complexity. Higher scores on the composite measure were significantly (*p* < .001) associated with larger response errors for any cognitive ability level (Tables 5 and 6 show “simple slopes” of item complexity for a cognitive test score of 0), and higher cognitive scores were associated with smaller response errors at any item complexity level (Tables 5 and 6 show simple slopes of cognitive ability for an item complexity score of 0). The interaction between item complexity and cognitive ability was significant (*p* < .001) in both samples. As shown in Fig. 2, the association between cognitive ability and response error scores became more pronounced as item complexity increased, supporting the TCH. Specifically, for each point increase in composite item complexity above 0 on the 0–9 scale, the relationship (regression slope) between cognitive ability and response error scores increased by 27% in the Wave 8 sample and by 16% in the Wave 9 sample.^1^

**Table 5:** Multilevel regression results for the prediction response error scores from cognitive test scores, item complexity, and their interaction, Wave 8 sample

	Complexity composite	WC	DC	UTT	VRT	VNP	CON	NEG	DIS	TEN	EXC
Fixed effects											
Intercept	41.97 (.25)	43.51 (.22)	45.09 (.22)	47.02 (.20)	44.76 (.22)	46.15 (.20)	45.50 (.21)	46.24 (.20)	45.75 (.21)	45.05 (.22)	46.82 (.21)
Item complexity	1.66 (.04)	7.66 (.19)	5.68 (.17)	1.38 (.15)	6.85 (.21)	6.21 (.20)	5.04 (.17)	7.12 (.22)	6.83 (.19)	5.30 (.17)	3.31 (.18)
Cognitive score	−.12 (.02)	−.14 (.01)	−.20 (.01)	−.23 (.01)	−.18 (.01)	−.21 (.01)	−.20 (.01)	−.20 (.01)	−.19 (.01)	−.18 (.01)	−.20 (.01)
Cognitive score × complexity	−.03 (.002)	−.17 (.01)	−.08 (.01)	−.003 (.01) ^a^	−.13 (.01)	−.09 (.01)	−.07 (.01)	−.20 (.01)	−.18 (.01)	−.11 (.01)	−.09 (.01)
Level 2 Random effects											
Intercept variance τ_00_	20.78	17.58	17.42	15.51	17.47	15.70	15.99	14.73	15.55	16.69	14.82
Slope variance τ_11_	.28	5.16	2.55	.19	6.89	.76	5.04	.36	6.83	2.01	1.57
Covariance τ_10_	−1.38	−4.11	−3.84	−1.58	−4.86	−2.43	−1.86	−.06	−1.78	−2.67	−.37
Level 1 σ^2^	281.64	285.03	287.48	292.51	285.53	289.18	289.07	290.61	289.51	289.14	291.65

Response error scores serving as dependent variable were transformed on a 0–100 scale (i.e., multiplied by 100) to avoid small numbers in the table. Values in parentheses are standard errors. All fixed effects coefficients are significant at *p* < .001, except ^a ^*p* = .75. WC = word count ≥10 words; DC = >1 word not in Dale–Chall word list; UTT = QUAID unfamiliar technical terms; VRT = QUAID vague or imprecise relative terms; VNP = QUAID vague or ambiguous noun phrases; CON = LIWC conjunctions; NEG = LIWC negations; DIS = LIWC discrepancy words; TEN = LIWC tentative statements; EXC = LIWC differentiation/exclusion words

**Table 6:** Multilevel regression results for the prediction response error scores from cognitive test scores, item complexity, and their interaction, Wave 9 sample

	Complexity composite	WC	DC	UTT	VRT	VNP	CON	NEG	DIS	TEN	EXC
Fixed effects											
Intercept	41.98 (.26)	43.72 (.23)	44.96 (.23)	47.18 (.21)	44.53 (.23)	46.26 (.21)	45.29 (.22)	46.20 (.21)	45.93 (.21)	45.06 (.23)	46.16 (.21)
Item complexity	1.66 (.04)	7.28 (.20)	6.02 (.19)	.73(.16)	7.53 (.21)	5.70 (.21)	5.67 (.19)	7.44 (.23)	6.11 (.20)	5.31 (.19)	3.46 (.19)
Cognitive score	−.16 (.02)	−.18 (.02)	−.22 (.01)	−.25 (.01)	−.21 (.01)	−.24 (.01)	−.23 (.01)	−.22 (.01)	−.22 (.01)	−.22 (.01)	−.23 (.01)
Cognitive score × complexity	−.03 (.002)	−.14 (.01)	−.09 (.01)	−.002 (.01) ^a^	−.12 (.01)	−.06 (.01)	−.06 (.01)	−.19 (.01)	−.14 (.01)	−.08 (.01)	−.07 (.01)
Level 2 Random effects											
Intercept variance τ_00_	23.54	19.45	19.00	15.97	17.47	16.82	17.45	15.67	16.26	18.38	15.91
Slope variance τ_11_	.35	6.21	3.91	.09	6.89	1.17	5.67	.37	0.69	3.27	2.22
Covariance τ_10_	−1.80	−5.48	−5.15	−1.01	−4.86	−3.94	−3.04	−.61	−1.69	−4.04	−1.00
Level 1 σ^2^	286.44	291.46	293.45	299.66	285.53	295.87	294.00	296.86	296.59	294.87	297.97

Response error scores serving as dependent variable were transformed on a 0–100 scale (i.e., multiplied by 100) to avoid small numbers in the table. Values in parentheses are standard errors. All fixed effects coefficients are significant at *p* < .001, except ^a ^*p* = .87. WC = word count ≥10 words; DC = >1 word not in Dale–Chall word list; UTT = QUAID unfamiliar technical terms; VRT = QUAID vague or imprecise relative terms; VNP = QUAID vague or ambiguous noun phrases; CON = LIWC conjunctions; NEG = LIWC negations; DIS = LIWC discrepancy words; TEN = LIWC tentative statements; EXC = LIWC differentiation/exclusion words

Sensitivity analyses were conducted to explore potential nonlinearities in the extent to which the continuous composite item complexity measure moderated the relationship between cognitive ability and response error scores. To this end, we entered the composite complexity measure as a categorical (rather than continuous) predictor in the moderated multilevel regression models. To facilitate model convergence (with multiple correlated random regression slopes for the effects of the complexity categories on response error scores), the categories of the composite complexity measure were entered as five bins, where bin 1 consisted of complexity scores 0–1, bin 2 of scores 2–3, bin 3 of scores 4–5, bin 4 of scores 6–7, and bin 5 of scores 8–9. Compared to the relationship (regression slope) between cognitive ability and response error scores for the bin with the lowest item complexity (i.e., bin 1), the relationships increased by 64% (for bin 2), 207% (bin 3), 232% (bin 4), and 323% (bin 5) in the Wave 8 sample, and by 65% (bin 2), 96% (bin 3), 122% (bin 4), and 198% (bin 5) in the Wave 9 sample, suggesting approximately linear increases across item complexity bins.

Results for the secondary moderated multilevel regression analyses conducted for each of the individual indicators of item complexity are also shown in Tables 5 and 6. In both samples, the cognitive ability by item complexity interaction term was significant (*p* < .001) for 9 of 10 individual indicators in the expected direction. The numerically strongest moderation effects were evident for items containing 10 or more words, items containing negations, and items containing discrepancies, the presence of which increased the association (regression slope) between cognitive ability and response error scores by 118%, 103%, and 94% in the Wave 8 sample and by 77%, 85%, and 61% in the Wave 9 sample. The exception was the index of unfamiliar technical terms, which did not significantly moderate the relationship between cognitive ability and response errors (*p* = .75 for Wave 8 and *p* = .87 for Wave 9 sample, respectively).

Given that the 102 PLQ items examined were included in 21 scales that may differ in overall item complexity, we also explored the correlations between cognitive ability scores and people’s average response errors on a scale-by-scale basis. The mean item composite complexities ranged from 0.00 to 6.33 across the 21 scales. The correlations between the cognitive ability scores and people’s average response errors ranged from *r = *−.228 to *r* = .115 across scales in Wave 8, and from *r* = −.257 to *r* = .137 in Wave 9. Differences in the mean item complexity between scales were significantly negatively associated with the magnitude of correlations between cognitive ability and response errors for corresponding scales, *r* = −.500 (95% CI = −.759 to −.070) for Wave 8 and *r* = −.478 (95% CI = −.748 to −.046) for Wave 9, respectively, indicating that response errors in scales comprising more complex items showed a stronger negative relationship with cognitive ability than response errors in scales with less complex items (see Figure S1 in the online appendix).

**Fig. 3:**
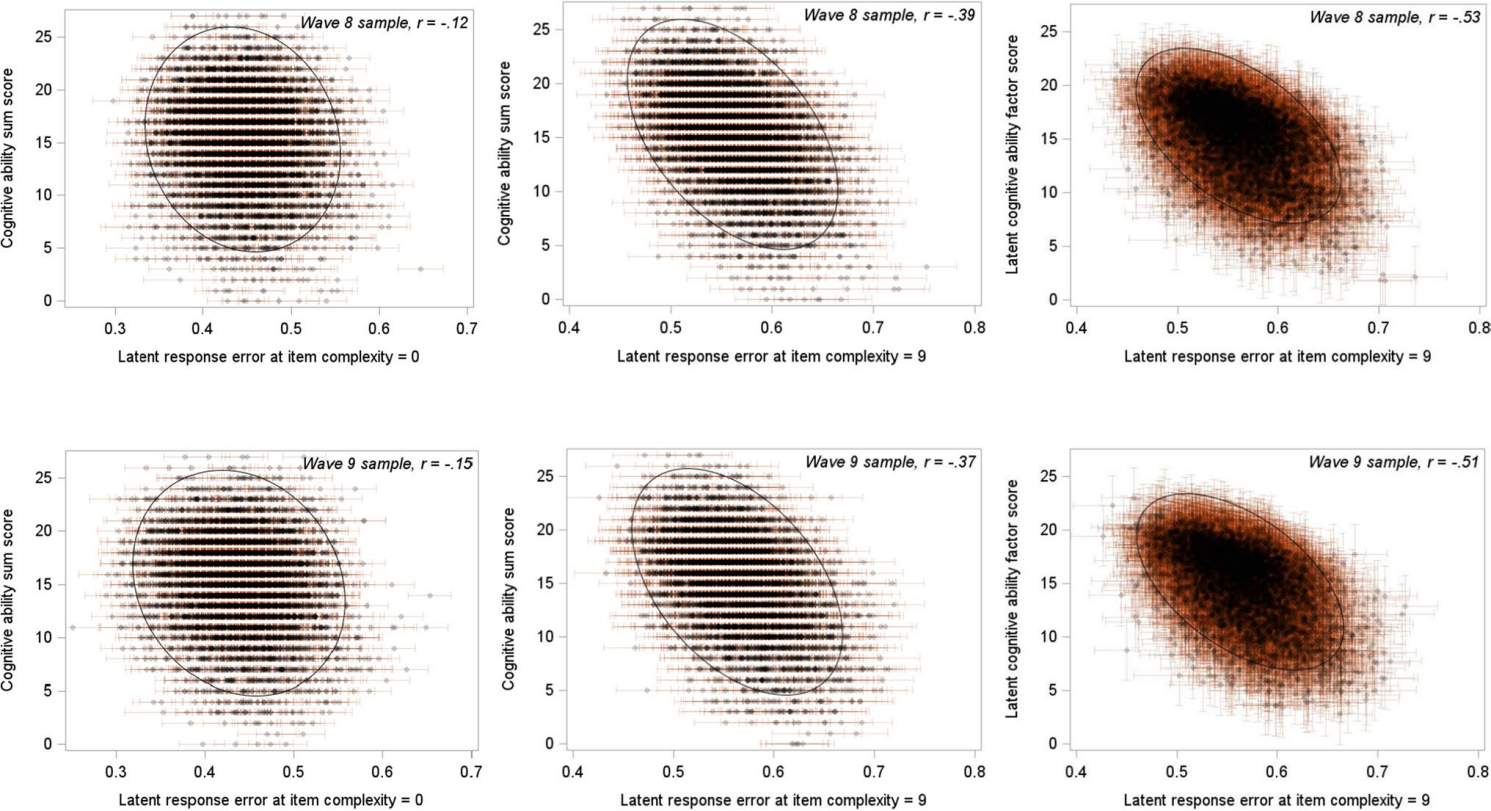
Scatter plots of the relationships between latent response errors at varying item complexity levels and cognitive ability scores in the Wave 8 (upper panel) and Wave 9 (lower panel) samples. Latent response errors are shown for item complexity levels of 0 (left panel) and 9 (middle and right panel). Cognitive ability scores are manifest sum scores (left and middle panel) and latent factor scores (right panel). Error bars represent standard errors for the factor scores of latent variables

The final analyses examined the correlations between people’s cognitive ability scores and latent variables representing individual differences in the magnitude of response errors for less versus more complex questions. As shown in Fig. 3 (left panel), the correlations between the cognitive ability sum score and latent response errors at low composite item complexity of 0 were *r* = −.115 (95% CI = −.142 to −.087; Wave 8 sample) and *r* = −.145 (95% CI = −.173 to −.118; Wave 9 sample). By contrast, the correlations between the cognitive ability sum score and latent response errors at high item complexity of 9 (middle panel in Fig. 3) were *r* = −.391 (95% CI = −.419 to −.367; Wave 8 sample) and *r* = −.374 (95% CI = −.403 to −.345; Wave 9 sample).^2^

The correlations between a *latent* cognitive ability factor and latent response errors were also estimated. A cognitive ability factor comprising the four cognitive subtests demonstrated adequate model fit in the Wave 8 sample (goodness of fit χ^2^ [*df* = 1] = 1.14, *p* = .28; CFI = 1.0, TLI = 1.0, RMSEA = .004, SRMR = .002) and in the Wave 9 sample (χ^2^ [*df* = 1] = 0.01, *p* = .91; CFI = 1.0, TLI = 1.0, RMSEA = .001, SRMR = .001). The correlations between the latent cognitive factor and latent response errors at low item complexity of 0 were *r* = −.154 (95% CI = −.192 to −.116; Wave 8 sample) and *r* = −.199 (95% CI = −.236 to −.163; Wave 9 sample). By contrast, the correlations between the latent cognitive factor and latent response errors at high item complexity of 9 were *r* = −.532 (95% CI = −.571 to −.493; Wave 8 sample) and *r* = −.513 (95% CI = −.554 to −.473; Wave 9 sample); see Fig. 3 (right panel).^3^

### Replication in subsequent HRS waves

The replication analyses involved repeated assessments of the same questionnaires in subsequent HRS waves. This allowed us to examine the long-term (4- and 8-year) retest correlations of the response error scores derived from the same samples. The mean retest correlation of respondents’ average response error scores across items was *r* = .625 (range = .607 to .646) for 4-year and *r* = .546 (range = .525 to .567) for 8-year intervals between assessment waves, respectively. Table S2 in the online appendix shows retest correlations involving the mean response error scores in each of the decile bins used for the analyses of the WPR as well as the response error scores estimated for less versus more complex questions; response errors in lower deciles showed somewhat lower retest correlations compared to those in the highest deciles (decile 1: mean *r* = .480 for 4-year and *r* = .417 for 8-year intervals; decile 10: mean *r* = .580 for 4-year and *r* = .505 for 8-year intervals).

Analyses of estimated response error scores and their relationships with cognitive ability scores in subsequent waves of HRS data yielded results that were almost identical to those in the Wave 8 and 9 samples and closely replicated the expected patterns based on the WPR and TCH. Details are shown in the online appendix (Tables S3–S7).

## Discussion

The idea that people’s patterns of performance across a series of trials of cognitively demanding tasks can reveal important aspects of their cognitive abilities has a long history and has been extensively pursued in research on reaction time tasks (Kyllonen & Zu, 2016; Schmiedek et al., 2007). The WPR and TCH are widely replicated in reaction time research and have been regarded as pointing to universal basic mental processes underlying individual differences in cognitive ability (Jensen, 2006; Schubert, 2019). The present study results provide robust support for the hypothesis that predictions by the WPR and TCH translate to patterns of response errors derived from questionnaires, highlighting that low-quality survey response patterns should not routinely be viewed as a lack of respondent effort for the task, but instead suggesting the possibility that people’s questionnaire response patterns may serve as an indirect indicator of cognitive ability in survey research.

As predicted by the WPR, when response error scores estimated from the 102 PLQ item responses were divided into decile bins based on each person’s distribution of error scores, we found the strongest associations of cognitive ability with a person’s *largest* response errors, whereas a person’s *smallest* response errors were virtually uncorrelated with cognitive ability. This is in line with the idea that the largest response error scores estimated from a GRM represent an individual’s worst performance on a questionnaire and that these error scores reveal more about cognitive ability than do other portions of the response error distribution. The largest response errors (worst performance) showed correlations with cognitive ability ranging from *r* = −.33 to *r* = −.35; an intriguing observation is that these correlations are nearly identical in magnitude to those obtained in a recent meta-analysis of the WPR in reaction time studies (Schubert, 2019), where general cognitive ability showed an overall correlation of *r* = −.33 with people’s slowest responses, suggesting close convergence across diverse task domains (response errors in questionnaires versus reaction times). Additionally, response error scores in the higher decile bins showed somewhat greater long-term retest reliability than response error scores in lower decile bins, suggesting that people’s worst performance on questionnaires is temporally more stable than their average or best performance.

To date, the exact psychological, cognitive, or biological processes underlying the WPR are still speculative. Conceptual frameworks that have been proposed in the reaction time literature include the attentional control account and the drift–diffusion model account of the WPR (see Coyle, 2003; Schmiedek et al., 2007; Schubert, 2019). According to the attentional control account, the WPR can be attributed to attentional variability and occasional lapses in sustained attention. Attentional lapses disrupt the representation and maintenance of task-relevant information in working memory, and they are thought to occur more frequently and to be more pronounced in people with lower cognitive abilities (Jensen, 1992; Larson & Alderton, 1990; Welhaf et al., 2020). The drift–diffusion model is a mathematical model for two-choice decision-making processes (Ratcliff & Rouder, 1998). One essential parameter in the diffusion model is the “drift rate” parameter, which captures the rate at which individuals accumulate information necessary for decision-making, and which has been assumed to reflect individual differences in general information-processing efficiency (Schmiedek et al., 2007). Consistent with the WPR, studies have shown that the drift rate strongly affects the shape of the distribution of an individual’s response times, in that it impacts the worst performance (longest reaction times) more than average or best performance (Ratcliff et al., 2008).

To what extent the same mechanisms underlying the WPR for reaction times on elementary cognitive tasks translate to response errors in questionnaires is currently unknown. However, Coyle (2001) found that the WPR applies to measures of performance accuracy in a strategic memory task, extending beyond reaction times in elementary cognitive tasks. Coyle (2001) suggested that neural transmission errors may result in general cognitive slowing and occasional cognitive disruptions, resulting in more pronounced dips in task performance. In the context of a person completing a questionnaire, deficits in attentional control and/or information-processing efficiency may similarly lead to more pronounced fluctuations in response quality and a higher rate of responses that deviate substantially from the statistically expected response.

Our findings also showed robust support for the predictions of the TCH, with high consistency across the analyzed samples and measurement waves. Response error scores for questions that were coded as overall more complex were much more strongly associated with cognitive ability than those for less complex questionnaire items. This finding corresponds with the idea that more complex items or tasks with a greater information load require more cognitive effort and are more likely to strain a person’s working memory, such that they have greater potential to clearly differentiate between individuals with higher and lower cognitive ability (Jensen, 2006). In secondary analyses that examined each aspect of item complexity individually, nearly all individual item complexity indicators showed the same pattern of results (with variation in the magnitude of effects) while being moderately correlated with each other, suggesting that the individual indicators tapped partially overlapping and partially complementary aspects of complexity that may contribute to the overall information load associated with each item.

There are limits to the expectation that the association between people’s cognitive ability and their performance steadily increases for progressively more complex tasks. That is, at some level of complexity, there necessarily is a turning point after which further complexity increases yield *lower* associations with cognitive ability as the cognitive load of the task approaches or exceeds the capacities of many individuals to perform well (see Lindley et al., 1995). Our sensitivity analyses did not yield evidence of such a “turning point” but rather suggested approximately linear increases in the association between cognitive ability and response error scores for increasing item complexity levels. To what extent this finding generalizes to other surveys beyond those in the present study is an open question. It is possible that the rating scale items examined in this study fell within a relatively narrow complexity range, or that the information load in most self-report questions is generally at a level low enough that this turning point is rarely reached or exceeded.

The present evidence has direct implications for researchers interested in obtaining an indirect indicator of participants’ cognitive abilities from self-report surveys. To extract information that is indicative of people’s cognitive ability level, one strategy is to select each person’s largest response error scores (i.e., their worst performance), or to extract response error scores specifically from selected items or scales with a higher average item complexity level. Another strategy is to estimate individual differences in response errors that are predicted for a relatively high item complexity level using the multilevel modeling procedures outlined above (see Eq. 7a and 7b) and based on all items administered. We think that the latter strategy may be preferrable because it does not discard items and because it statistically controls for the heterogeneity in complexity levels across items. A working example with step-by-step instructions and annotated software code is available at https://osf.io/vja3t/ for readers who wish to apply the required procedures to their own data. Our results showed that a latent variable of response errors estimated for the most complex PLQ items correlated at about .50 with a latent cognitive ability factor, suggesting that this strategy yields an individual differences measure that shares about 25% of the variance with people’s true cognitive ability.

## Limitations and directions for future research

This study has several limitations that should be noted. First, even though we found robust effects supporting the WPR and TCH that were replicated in two independent samples and across multiple waves of a large longitudinal aging study, the two samples were drawn from the same population of older adults in the United States who participated in the HRS, and the same questionnaire items were administered to all respondents. Additional research is required to examine whether the current findings generalize to younger respondent samples and to surveys with different self-report questions.

Second, although the use of IRT provided a theoretically sound basis for assessing the quality of individual item responses, the method rests on the assumption that the model fits the data well and that the IRT model parameters are themselves not excessively biased by overall low response quality. We applied a simple unidimensional GRM to all 21 PLQ scales, which yielded a well-fitting model for many scales but also showed expected variation in model fit statistics as the PLQ scales were not originally developed using IRT principles. To improve the estimation of response error scores from IRT models, future research could consider the utility of multidimensional IRT models fitted to the items of multiple scales simultaneously, as well as iterative scale purification procedures that have been shown to reduce potential bias in the estimation of GRM item parameters when some respondents have overall low response quality (Hong & Cheng, 2019; Qiu et al., 2024). Moreover, our IRT modeling strategy implicitly assumed that the same measurement model used to assess the constructs underlying the PLQ scales holds equally well for all people. In fact, individuals or participant subgroups may use different implicit theories about the constructs assessed or may interpret specific items on a scale differently. This may have confounded the measurement of response error scores with actual differences in the way people’s true scores on a construct related to the probability of their responses. Tests for measurement invariance and adjustments for differential item functioning (Zumbo, 2007) applied to each scale before estimating response error scores could be used to reduce this potential confound.

Third, the cognitive measures in the HRS were designed with a focus on the detection of cognitive deficits at older ages (Crimmins et al., 2016). Even though it has been acknowledged that there is no universally optimal way to define the constituents of general cognitive ability or intelligence (van der Maas et al., 2014), the specific composition of cognitive subtests in the HRS may have impacted the results. In future research, it will be important to replicate the present findings with a different composition of cognitive tests to evaluate the generalizability of the present results. Moreover, it will be beneficial to examine data from studies that assess a broader range of cognitive domains than that available in the HRS data analyzed here to better understand whether specific cognitive functions are more closely related to response errors in questionnaires than other cognitive functions.

Fourth, even though we included multiple indicators of item complexity in our analysis, they were not without limitations. We considered only aspects of the questions themselves and did not code the complexity of the response scales, because the items in the PLQ consistently used ordinal response scales with little variation in response format and number of response options. We also relied exclusively on complexity indicators that can be automatically derived from text analysis software (QUAID, Graesser et al., 2000; LIWC, Pennebaker et al., 2015). Potentially important aspects of information load that require judgment by human coders (e.g., to what extent answering a question involves retrieval of information from memory) were not included, as these have been shown to suffer from low inter-coder reliability (Bais et al., 2019). More work is required to develop an optimal (composite) measure of question complexity.

Fifth, the present study was limited to questionnaires administered in paper-and-pencil format. Self-report surveys are increasingly administered electronically over the internet, and the results should be replicated with responses from web-administered surveys. Web-based data collection also provides access to additional sources of survey response behaviors that were not considered here, including item response latencies recorded passively as paradata alongside the actual item responses, which have previously proven useful as indicators of people’s cognitive ability (Junghaenel et al., 2023; Schneider, Junghaenel et al., 2023b).

Finally, our analyses did not consider changes in response errors over the course of the questions within a survey and across multiple repeated assessment waves. Within a given survey, respondents with lower cognitive ability may be especially prone to lapses in concentration (with a possible increase in the likelihood of larger response errors) toward the end of the survey and after having expended potentially significant amounts of cognitive effort on previous survey questions (see Bowling et al., 2021). Across multiple assessment waves, participants are repeatedly exposed to the same questions, which may increase response quality (a possible decrease in response errors) due to practice effects and increasing familiarity with the questions (Kartsounidou et al., 2023), and the extent to which people benefit from practice may itself be a marker of individual differences in cognitive ability (Jensen, 2006; Jutten et al., 2020; Minear et al., 2018). Examining these dynamics is an interesting avenue for future research in that this could facilitate the development of strategies to further augment the usefulness of questionnaire response patterns as indirect cognitive ability indicators.

## Conclusions

The present study results support the idea that response patterns in questionnaires reveal meaningful information about individual differences in general cognitive ability and provide new strategies for developing indirect indicators of questionnaire response quality that are most closely associated with people’s cognitive ability levels. Even though indirect performance indicators derived from survey response patterns are not a surrogate for formal cognitive tests, and should not be viewed as such, our results suggest that they might supplement cognitive test scores or serve as a rough indicator to examine group differences in cognitive ability in survey studies that have no cognitive test data available.

### Supplementary Information

Below is the link to the electronic supplementary material.

Supplementary file1 (DOCX 56 KB)

## Footnotes

1 The percentage differences in the relationship (regression slope) between cognitive ability and response error scores per point increase in item complexity were computed as 100 * (interaction term / regression slope of cognitive ability for an item complexity score of 0).

2 We also estimated the correlations between the cognitive ability sum score and the latent response errors for other item complexity levels by centering the continuous item complexity predictor variable at intermediate levels in the multilevel model. The resulting correlations for integer item complexity scores of 1 to 8 were −.155, −.197, −.240, −.281, −.317, −.346, −.368, −.382 (Wave 8 sample) and −.182, −.220, −.259, −.296, −.327, −.350, −.365, −.371 (Wave 9 sample), respectively.

3 Correlations for integer item complexity scores of 1 to 8 were −.209, −.267, −.326, −.381, −.430, −.470, −.500, −.520 (Wave 8 sample) and −.249, −.302, −.356, −.406, −.448, −.480, −.501, −.511 (Wave 9 sample), respectively.
