# Supplementary material for: Can you tell people’s cognitive ability level from their response patterns in questionnaires?
Source: Behav Res Methods. 2024 Mar 25;56(7):6741–58. doi: 10.3758/s13428-024-02388-2 (PMC11362444; doi:10.3758/s13428-024-02388-2)
Supplement: Supplementary file 1 — Supplementary file1 (DOCX 56 KB) [file 13428_2024_2388_MOESM1_ESM.docx]

**Supplemental Online Appendix**

**Can you tell people’s cognitive ability level from their response patterns in questionnaires?**

Stefan Schneider, Raymond Hernandez, Doerte U. Junghaenel, Haomiao Jin, Pey-Jiuan Lee, Hongxin Gao, Danny Maupin, Bart Orriens, Erik Meijer, Arthur A. Stone

Table S1: Fit of Graded Response Models for each of the Psychosocial scales in the Participant Lifestyle Questionnaire

|  | *N* | Response | Wave 8 sample | | | |  | | Wave 9 sample | | | |
| --- | --- | --- | --- | --- | --- | --- | --- | --- | --- | --- | --- | --- |
| Scale | items | options | CFI | TLI | RMSEA | SRMR | | CFI | | TLI | RMSEA | SRMR |
| Life satisfaction | 5 | 6/7^a^ | .988 | .975 | .143 | .034 | | .988 | | .975 | .152 | .036 |
| Cynical hostility | 5 | 6 | .953 | .906 | .135 | .035 | | .947 | | .894 | .137 | .039 |
| Optimism | 6 | 6 | .874 | .790 | .237 | .152 | | .879 | | .798 | .226 | .139 |
| Hopelessness | 4 | 6 | .999 | .998 | .041 | .003 | | .981 | | .943 | .198 | .030 |
| Loneliness | 3 | 3 | 1.00 | 1.00 | .000 | .000 | | 1.00 | | 1.00 | .000 | .000 |
| Neighborhood physical disorder | 4 | 7 | .970 | .911 | .103 | .019 | | .993 | | .979 | .086 | .011 |
| Neighborhood social cohesion | 4 | 7 | .997 | .991 | .055 | .005 | | .997 | | .992 | .075 | .008 |
| Constraints on personal control | 5 | 6 | .989 | .978 | .095 | .020 | | .987 | | .974 | .114 | .026 |
| Perceived mastery | 5 | 6 | .979 | .959 | .195 | .067 | | .984 | | .969 | .188 | .063 |
| Religiosity/Spirituality | 4 | 6 | .996 | .988 | .196 | .029 | | .996 | | .988 | .184 | .027 |
| Everyday discrimination | 5 | 6 | .975 | .951 | .099 | .038 | | .980 | | .961 | .092 | .035 |
| Social effort/reward balance | 3 | 5 | 1.00 | 1.00 | .000 | .000 | | 1.00 | | 1.00 | .000 | .000 |
| Extraversion | 5 | 4 | .974 | .921 | .132 | .036 | | .972 | | .916 | .134 | .039 |
| Agreeableness | 5 | 4 | .985 | .971 | .054 | .019 | | .991 | | .982 | .042 | .016 |
| Neuroticism | 4 | 4 | .985 | .971 | .054 | .019 | | .957 | | .915 | .114 | .052 |
| Conscientiousness | 5 | 4 | .980 | .959 | .052 | .020 | | .978 | | .956 | .054 | .021 |
| Openness to experience | 7 | 4 | .933 | .900 | .106 | .068 | | .936 | | .904 | .118 | .077 |
| Purpose in life | 7 | 6 | .868 | .803 | .121 | .064 | | .882 | | .823 | .122 | .064 |
| Anxiety | 5 | 4 | .970 | .939 | .115 | .053 | | .974 | | .947 | .115 | .051 |
| Anger-in | 4 | 4 | .972 | .915 | .157 | .032 | | .959 | | .878 | .202 | .047 |
| Anger-out | 7 | 4 | .975 | .963 | .085 | .080 | | .974 | | .961 | .088 | .081 |

*Note*: For details on each scale, see Smith J, Fisher GG, Ryan L, Clarke P, House J, Weir D. *Health and Retirement Study Psychosocial and Lifestyle Questionnaire 2006 - 2010: Documentation Report.* Ann Arbor, MI: University of Michigan; 2013.

^a^ Administered using a 6-point scale in Wave 8 and a 7-point scale in Wave 9.

Figure S1: Scatterplots of correlations between scale mean response errors and cognitive ability scores (y-axis) plotted against the scale mean item complexity level (x-axis) of each of the scales in the Participant Lifestyle Questionnaire. The upper panel shows the plot for the Wave 8 sample and the lower panel shows the plot for the Wave 9 sample.

Table S2: Long-term (4- and 8-year) retest correlations between response errors across assessment waves in the Health and Retirement Study (HRS)

|  | *HRS participant sample completing the PLQ in Waves 8, 10, and 12 (years 2006, 2010, and 2014)* | | |  | *HRS participant sample completing the PLQ in Waves 9, 11, and 13 (years 2008, 2012, and 2016)* | | |
| --- | --- | --- | --- | --- | --- | --- | --- |
|  | **Correlations** | | |  | **Correlations** | | |
|  | 2006 with 2010 | 2010 with 2014 | 2006 with 2014 |  | 2008 with 2012 | 2012 with 2016 | 2008 with 2016 |
| Mean response error across all items | .639 | .607 | .525 |  | .646 | .607 | .567 |
| Mean response error for each decile of a person’s distribution of errors |  |  |  |  |  |  |  |
| Decile 1 | .524 | .452 | .399 |  | .504 | .439 | .434 |
| Decile 2 | .550 | .508 | .448 |  | .550 | .502 | .476 |
| Decile 3 | .570 | .528 | .462 |  | .579 | .528 | .495 |
| Decile 4 | .580 | .546 | .479 |  | .584 | .545 | .513 |
| Decile 5 | .585 | .559 | .487 |  | .592 | .571 | .527 |
| Decile 6 | .600 | .563 | .491 |  | .602 | .576 | .531 |
| Decile 7 | .598 | .566 | .488 |  | .611 | .575 | .535 |
| Decile 8 | .605 | .577 | .507 |  | .617 | .589 | .535 |
| Decile 9 | .610 | .580 | .508 |  | .618 | .587 | .543 |
| Decile 10 | .594 | .555 | .494 |  | .595 | .574 | .516 |
| Response errors for items with lower versus higher complexity levels |  |  |  |  |  |  |  |
| Complexity level = 0 | .633 | .595 | .527 |  | .642 | .595 | .550 |
| Complexity level = 9 | .576 | .539 | .482 |  | .543 | .535 | .483 |

Note: PLQ = Personality and Lifestyle Questionnaire

Table S3: Correlations (95% confidence intervals) between cognitive test scores and mean response error scores across deciles in Wave 10 to Wave 13 samples.

|  | Correlation with cognitive test scores | | | |
| --- | --- | --- | --- | --- |
|  | Wave 10 sample | Wave 11 sample | Wave 12 sample | Wave 13 sample |
| Response errors decile 1 | -.03 (-.05; -.01) ^a^ | -.01 (-.04; .01) ^b^ | -.02 (-.04; .01) ^c^ | -.03 (-.06; -.01) ^a^ |
| Response errors decile 2 | -.09 (-.11; -.06) | -.07 (-.09; -.04) | -.07 (-.09; -.05) | -.07 (-.10; -.05) |
| Response errors decile 3 | -.11 (-.13; -.09) | -.09 (-.12; -.07) | -.10 (-.12; -.07) | -.10 (-.12; -.07) |
| Response errors decile 4 | -.14 (-.16; -.12) | -.13 (-.15; -.11) | -.13 (-.16; -.11) | -.13 (-.15; -.11) |
| Response errors decile 5 | -.17 (-.19; -.15) | -.16 (-.18; -.14) | -.17 (-.19; -.15) | -.16 (-.18; -.14) |
| Response errors decile 6 | -.21 (-.23; -.19) | -.20 (-.22; -.18) | -.21 (-.23; -.19) | -.20 (-.23; -.18) |
| Response errors decile 7 | -.24 (-.26; -.22) | -.23 (-.25; -.21) | -.24 (-.27; -.22) | -.23 (-.26; -.21) |
| Response errors decile 8 | -.28 (-.30; -.25) | -.26 (-.28; -.24) | -.27 (-.29; -.25) ^d^ | -.26 (-.28; -.24) |
| Response errors decile 9 | -.31 (-.33; -.29) | -.30 (-.32; -.27) | -.29 (-.31; -.27) ^e^ | -.28 (-.30; -.26) ^d^ |
| Response errors decile 10 | -.34 (-.36; -.32) | -.33 (-.35; -.31) | -.29 (-.31; -.27) ^de^ | -.30 (-.32; -.28) ^d^ |
| Wald test for differences in correlations across all deciles | χ^2^(*df* = 9) = 488.57 *p* < .001 | χ^2^(*df* = 9) = 476.81 *p* < .001 | χ^2^(*df* = 9) = 388.91 *p* < .001 | χ^2^(*df* = 9) = 332.32 *p* < .001 |

*Note*: All correlation coefficients are significant at *p* <.001 except ^a^ *p* <.01, ^b^ *p* = .28, ^c^ *p* = .19. All correlations significantly differ from each other between all deciles in each sample (*p* < .001), except ^d^ *p* = .02, ^e^ *p* = .99.

Table S4. Multilevel regression results for the prediction response error scores from cognitive test scores, item complexity, and their interaction, Wave 10 sample.

|  | Complexity composite | WC | DC | UTT | VRT | VNP | CON | NEG | DIS | TEN | EXC |
| --- | --- | --- | --- | --- | --- | --- | --- | --- | --- | --- | --- |
| Fixed effects |  |  |  |  |  |  |  |  |  |  |  |
| Intercept | 42.14  (.25) | 43.84 (.23) | 45.04 (.22) | 47.17 (.20) | 44.46 (.22) | 46.35 (.21) | 45.47 (.21) | 46.12 (.20) | 45.94 (.20) | 45.16 (.22) | 46.25 (.21) |
| Item   complexity | 1.63  (.06) | 6.98 (.20) | 5.76 (.18) | 0.69 (.15) | 7.63 (.21) | 5.18 (.20) | 5.10 (.17) | 7.84 (.22) | 5.99 (.19) | 5.03 (.18) | 3.15 (.18) |
| Cognitive   score | -.16  (.02) | -.15 (.01) | -.21 (.01) | -.25 (.01) | -.15 (.01) | -.24 (.01) | -.23 (.01) | -.22 (.01) | -.21 (.01) | -.21 (.01) | -.23 (.01) |
| Cognitive  score X  complexity | -.03  (.002) | -.18 (.01) | -.10 (.01) | -.005 (.01) ^a^ | -.20 (.01) | -.05 (.01) | -.05 (.01) | -.23 (.01) | -.15 (.01) | -.09 (.01) | -.06 (.01) |
| Level 2  random effects |  |  |  |  |  |  |  |  |  |  |  |
| Intercept   variance τ_00_ | 22.92 | 19.01 | 18.75 | 16.08 | 19.17 | 16.85 | 17.34 | 15.72 | 16.36 | 18.17 | 15.95 |
| Slope   variance τ_11_ | .31 | 5.71 | 3.41 | .11 | 7.59 | 1.11 | 5.10 | .40 | .83 | 2.38 | 1.57 |
| Covariance   τ_10_ | -1.63 | -4.90 | -4.71 | -1.17 | -6.19 | -3.47 | -2.66 | -.65 | -1.85 | -3.56 | -.89 |
| Level 1 σ^2^ | 281.92 | 286.05 | 287.92 | 293.05 | 284.54 | 289.94 | 288.59 | 290.46 | 290.44 | 289.28 | 291.62 |

*Note*: Response error scores serving as dependent variable were transformed on a 0-100 scale (i.e., multiplied with 100) to avoid small numbers in the table. Values in parentheses are standard errors. All fixed effects coefficients are significant at *p* <.001, except ^a^ *p* = .61. WC = Word count ≥10 words; DC = >1 word not in Dale-Chall word list; UTT = QUAID unfamiliar technical terms; VRT = QUAID vague or imprecise relative terms; VNP = QUAID vague or ambiguous noun phrases; CON = LIWC conjunctions; NEG = LIWC negations; DIS = LIWC discrepancy words; TEN = LIWC tentative statements; EXC = LIWC differentiation/exclusion words.

Table S5. Multilevel regression results for the prediction response error scores from cognitive test scores, item complexity, and their interaction, Wave 11 sample.

|  | Complexity composite | WC | DC | UTT | VRT | VNP | CON | NEG | DIS | TEN | EXC |
| --- | --- | --- | --- | --- | --- | --- | --- | --- | --- | --- | --- |
| Fixed effects |  |  |  |  |  |  |  |  |  |  |  |
| Intercept | 41.79  (.27) | 43.51 (.24) | 44.61 (.23) | 46.75 (.21) | 44.10 (.24) | 46.01 (.22) | 45.12 (.23) | 45.77 (.21) | 45.39 (.22) | 44.93 (.23) | 45.90 (.22) |
| Item   complexity | 1.59  (.04) | 6.84 (.20) | 5.85 (.19) | .80  (.16) | 7.58 (.22) | 4.89 (.20) | 4.96 (.18) | 7.53 (.23) | 6.72 (.20) | 4.66 (.18) | 3.01 (.18) |
| Cognitive   score | -.14  (.02) | -.16 (.02) | -.20 (.02) | -.24 (.01) | -.18 (.02) | -.23 (.01) | -.21 (.01) | -.20 (.01) | -.20 (.01) | -.20 (.02) | -.22 (.01) |
| Cognitive  score X  complexity | -.03  (.003) | -.15 (.01) | -.11 (.01) | .007 (.01) ^a^ | -.15 (.01) | -.03 (.01) ^b^ | -.06 (.01) | -.22 (.01) | -.16 (.01) | -.08 (.01) | -.06 (.01) |
| Level 2  random effects |  |  |  |  |  |  |  |  |  |  |  |
| Intercept   variance τ_00_ | 25.12 | 20.78 | 20.23 | 17.38 | 20.93 | 18.15 | 18.86 | 17.34 | 18.43 | 19.61 | 17.52 |
| Slope   variance τ_11_ | .32 | 6.36 | 3.55 | .10 | 8.43 | .89 | 1.09 | .23 | 6.72 | 2.62 | 1.50 |
| Covariance   τ_10_ | -1.79 | -5.46 | -4.89 | -1.07 | -6.91 | -3.39 | -2.84 | -1.54 | -3.51 | -3.69 | -1.18 |
| Level 1 σ^2^ | 286.69 | 291.12 | 292.69 | 297.79 | 289.18 | 294.74 | 293.89 | 295.47 | 294.25 | 294.41 | 296.58 |

*Note*: Response error scores serving as dependent variable were transformed on a 0-100 scale (i.e., multiplied with 100) to avoid small numbers in the table. Values in parentheses are standard errors. All fixed effects are significant at *p* <.001, except ^a^ *p* = .50 and ^b^*p* = .01. WC = Word count ≥10 words; DC = >1 word not in Dale-Chall word list; UTT = QUAID unfamiliar technical terms; VRT = QUAID vague or imprecise relative terms; VNP = QUAID vague or ambiguous noun phrases; CON = LIWC conjunctions; NEG = LIWC negations; DIS = LIWC discrepancy words; TEN = LIWC tentative statements; EXC = LIWC differentiation/exclusion words.

Table S6. Multilevel regression results for the prediction response error scores from cognitive test scores, item complexity, and their interaction, Wave 12 sample.

|  | Complexity composite | WC | DC | UTT | VRT | VNP | CON | NEG | DIS | TEN | EXC |
| --- | --- | --- | --- | --- | --- | --- | --- | --- | --- | --- | --- |
| Fixed effects |  |  |  |  |  |  |  |  |  |  |  |
| Intercept | 41.87  (.27) | 43.32 (.25) | 44.82 (.24) | 46.57 (.23) | 44.38 (.25) | 45.63 (.23) | 45.07 (.23) | 45.44 (.22) | 45.18 (.23) | 44.82 (.23) | 45.58 (.22) |
| Item   complexity | 1.59  (.05) | 7.36 (.23) | 5.09 (.21) | .32  (.18) | 6.41 (.23) | 5.64 (.20) | 5.10 (.21) | 8.25 (.25) | 5.33 (.21) | 4.85 (.21) | 3.48 (.22) |
| Cognitive   score | -.16  (.02) | -.18 (.02) | -.21 (.02) | -.25 (.01) | -.25 (.02) | -.25 (.01) | -.21 (.01) | -.23 (.01) | -.21 (.01) | -.21 (.01) | -.22 (.01) |
| Cognitive  score X  complexity | -.03  (.003) | -.17 (.01) | -.12 (.01) | -.01 (.01) ^a^ | -.02 (.01) ^b^ | -.04 (.02) ^c^ | -.14 (.01) | -.16 (.01) | -.15 (.01) | -.12 (.01) | -.11 (.01) |
| Level 2  random effects |  |  |  |  |  |  |  |  |  |  |  |
| Intercept   variance τ_00_ | 22.27 | 19.81 | 18.99 | 18.40 | 21.73 | 19.23 | 17.01 | 18.05 | 17.85 | 17.95 | 16.56 |
| Slope   variance τ_11_ | 1.59 | 7.36 | .31 | .32 | 6.41 | 3.77 | 5.10 | .16 | 5.32 | .63 | 2.33 |
| Covariance   τ_10_ | -1.17 | -3.35 | -1.79 | -1.41 | -6.64 | -4.33 | 1.14 | -1.00 | -.24 | -.39 | 1.52 |
| Level 1 σ^2^ | 295.29 | 298.43 | 302.74 | 305.47 | 295.11 | 300.95 | 303.40 | 301.05 | 303.45 | 303.00 | 304.26 |

*Note*: Response error scores serving as dependent variable were transformed on a 0-100 scale (i.e., multiplied with 100) to avoid small numbers in the table. Values in parentheses are standard errors. All fixed effects are significant at *p* <.001, except ^a^ *p* = .22, ^b^ *p* = .14, ^c^*p* = .02. WC = Word count ≥10 words; DC = >1 word not in Dale-Chall word list; UTT = QUAID unfamiliar technical terms; VRT = QUAID vague or imprecise relative terms; VNP = QUAID vague or ambiguous noun phrases; CON = LIWC conjunctions; NEG = LIWC negations; DIS = LIWC discrepancy words; TEN = LIWC tentative statements; EXC = LIWC differentiation/exclusion words.

Table S7. Multilevel regression results for the prediction response error scores from cognitive test scores, item complexity, and their interaction, Wave 13 sample.

|  | Complexity composite | WC | DC | UTT | VRT | VNP | CON | NEG | DIS | TEN | EXC |
| --- | --- | --- | --- | --- | --- | --- | --- | --- | --- | --- | --- |
| Fixed effects |  |  |  |  |  |  |  |  |  |  |  |
| Intercept | 42.66  (.31) | 44.00 (.29) | 45.59 (.27) | 47.17 (.26) | 44.98 (.29) | 46.35 (.27) | 45.92 (.27) | 46.23 (.26) | 45.87 (.27) | 45.61 (.28) | 46.22 (.27) |
| Item   complexity | 1.65  (.05) | 7.39 (.25) | 4.85 (.24) | .66 (.21) | 6.66 (.26) | 5.51 (.30) | 4.58 (.24) | 7.54 (.28) | 5.32 (.23) | 4.57 (.24) | 3.59 (.25) |
| Cognitive   score | -.17  (.02) | -.19 (.02) | -.23 (.02) | -.26 (.02) | -.26 (.02) | -.27 (.02) | -.22 (.02) | -.24 (.02) | -.22 (.02) | -.22 (.02) | -.22 (.02) |
| Cognitive  score X  complexity | -.03  (.003) | -.17 (.02) | -.11 (.02) | -.02 (.01) ^a^ | -.03 (.02) ^a^ | -.01 (.02) ^b^ | -.15 (.02) | -.14 (.02) | -.16 (.01) | -.11 (.02) | -.14 (.02) |
| Level 2  random effects |  |  |  |  |  |  |  |  |  |  |  |
| Intercept   variance τ_00_ | 23.53 | 21.00 | 19.61 | 19.10 | 22.69 | 20.63 | 17.82 | 19.36 | 19.25 | 19.50 | 17.99 |
| Slope   variance τ_11_ | .33 | 5.59 | .92 | .66 | 6.40 | 4.76 | .62 | .13 | .19 | 1.88 | 3.59 |
| Covariance   τ_10_ | -1.26 | -3.53 | -1.17 | -.46 | -6.29 | -5.12 | 1.63 | -1.47 | -.67 | -1.15 | .91 |
| Level 1 σ^2^ | 285.39 | 288.11 | 292.47 | 295.25 | 284.75 | 290.09 | 293.89 | 291.49 | 293.40 | 292.81 | 293.92 |

*Note*: Response error scores serving as dependent variable were transformed on a 0-100 scale (i.e., multiplied with 100) to avoid small numbers in the table. Values in parentheses are standard errors. All fixed effects are significant at *p* <.001, except ^a^ *p* = .06 and ^b^ *p* = .62. WC = Word count ≥10 words; DC = >1 word not in Dale-Chall word list; UTT = QUAID unfamiliar technical terms; VRT = QUAID vague or imprecise relative terms; VNP = QUAID vague or ambiguous noun phrases; CON = LIWC conjunctions; NEG = LIWC negations; DIS = LIWC discrepancy words; TEN = LIWC tentative statements; EXC = LIWC differentiation/exclusion words.
